# Comparative analysis of mineralocorticoid receptor antagonists and renin-angiotensin system inhibitors/angiotensin receptor neprilysin inhibitor in heart failure with mildly reduced ejection fraction

**DOI:** 10.3389/fphar.2024.1507326

**Published:** 2024-12-13

**Authors:** Chia-Chen Lee, David Te-Wei Kuan, Kai-Chun Chang, Zheng-Wei Chen, Jen-Fang Cheng, Ting-Tse Lin, Lian-Yu Lin, Cho-Kai Wu

**Affiliations:** ^1^ Department of Internal Medicine, College of Medicine, National Taiwan University, Taipei, Taiwan; ^2^ Division of Cardiology, Department of Internal Medicine, Fu Jen Catholic University Hospital, New Taipei City, Taiwan; ^3^ Division of Cardiology, Department of Internal Medicine, National Taiwan University College of Medicine and Hospital Yunlin Branch, Yunlin, Taiwan; ^4^ Division of Cardiology, Department of Internal Medicine, National Taiwan University College of Medicine and Hospital, Taipei, Taiwan

**Keywords:** HFmrEF, cardiovascular outcomes, reninangiotensin system inhibitor, angiotensin receptor neprilysin inhibitor, MRA (magnetic resonance angiography)

## Abstract

**Introduction:**

To clarify the efficacy of mineralocorticoid receptor antagonists (MRA) and renin-angiotensin system inhibitors/angiotensin receptor neprilysin inhibitors (RASI/ARNI) in heart failure with mildly reduced ejection fraction (HFmrEF).

**Methods:**

This study assessed the association between these medications and outcomes in HFmrEF using data from the National Taiwan University Hospital-integrated Medical Database. The primary outcome was cardiovascular mortality/heart failure hospitalization (HHF). Inverse probability of treatment weighting balanced baseline patient characteristics. The exposure of primary interest was use of MRA and use of RASI/ARNI, while the non-user group was also likely to receive other heart failure medication treatment.

**Results:**

Among 2,584 HFmrEF patients, 17% received MRA and 43% received RASI/ARNI. Predictors of MRA use included older age, slightly higher ejection fraction, and lower NT-proBNP level. RASI/ARNI use was predicted by higher BMI, lower NT-proBNP level, normal uric acid and potassium levels. MRA use was not associated with a lower risk of cardiovascular death [hazard ratio = 0.89, 95% confidence interval (CI): 0.78–1.02] or HHF (hazard ratio = 1.01, 95% CI: 0.94–1.09). Conversely, RASI//ARNI use was linked to a lower risk of cardiovascular death (hazard ratio = 0.82, 95% CI: 0.71–0.94) but not HHF (hazard ratio = 0.995, 95% CI: 0.924–1.07). Landmark analysis showed no significant difference in outcomes for follow-up durations exceeding 2 years.

**Conclusion:**

MRA had a neutral effect on cardiovascular death and HHF, while RASI/ARNI was associated with a lower risk of cardiovascular death. RASI/ARNI may be more beneficial than MRA for HFmrEF patients. Regular re-evaluation is essential to adjust heart failure treatment.

## Introduction

Left ventricle ejection fraction (LVEF) is a predictor of adverse outcomes and medication effectiveness among patients with heart failure (HF) (Dimond et al., 2024). Measured by echocardiography, LVEF is central to HF diagnosis, with values between 41% and 49% indicating heart failure with mildly reduced ejection fraction (HFmrEF) (McDonagh et al., 2023a; Maddox et al., 2024). HFmrEF shares features with both heart failure with reduced ejection fraction (HFrEF) and preserved ejection fraction (HFpEF). Similar to HFrEF, HFmrEF is often associated with ischemic heart disease and less renal impairment, while it resembles HFpEF in milder symptoms, lower natriuretic peptide levels and better cardiovascular outcome (Tsuji et al., 2017a). In regard to comorbidity, the age and atrial fibrillation incidence of HFmrEF patients tend to be intermediate between those with HFrEF and HFpEF. Accordingly, HFmrEF is considered to be in an intermediate zone (McDonagh et al., 2021; Dimond et al., 2024).

Current guidelines only endorse SGLT2 inhibitors for HFmrEF treatment (Class I) (Solomon et al., 2022; McDonagh et al., 2023a; McDonagh et al., 2023b), while renin–angiotensin system inhibitors (RASI), angiotensin receptor-neprilysin inhibitor (ARNI), and mineralocorticoid receptor antagonist (MRA) remain a Class IIb recommendation (McDonagh et al., 2021), based on subgroup or *post hoc* analyses. The TOPCAT trial showed that spironolactone reduced HF hospitalizations in patients with an LVEF below 55% (Solomon et al., 2016a), while the PARAGON-HF trial found sacubitril/valsartan reduced cardiovascular death and HF hospitalizations by 22% in patients with EF ≤ 57%. Combined analysis of PARADIGM-HF and PARAGON-HF showed significant reduction in HF hospitalizations for HFmrEF patients treated with sacubitril/valsartan (Solomon et al., 2020).

However, many HFmrEF patients also have comorbidities as coronary artery disease (CAD), hypertension, or post-myocardial infarction systolic dysfunction, which might benefit from RASI/ARNI and MRA (McDonagh et al., 2021). A recent randomized controlled trial demonstrated that a new generation of nonsteroidal MRA finerenone led to positive cardiovascular outcome among patients with HFpEF and HFrEF (Solomon et al., 2024). Another recent large retrospective study reported favorable outcomes using RASI/ARNI and β-blockers in Western HFmrEF patients (Stolfo et al., 2023). However, there are limited dedicated trials specifically for RASI/ARNI/MRA in HFmrEF, especially in Asian populations. Our study aims to assess the use of MRA and RASI/ARNI, as well as patient demographics, and their associations with cardiovascular mortality, HF hospitalizations and all-cause mortality in a large, real-world Asian HFmrEF cohort.

## Materials and methods

### Study design and data sources

This large, observational, and retrospective multicenter cohort study spanned from 2016 to 2022. Demographic, diagnostic, and medical records were sourced from the National Taiwan University Hospital-integrative Medical Database (NTUH-iMD). Informed consent was not required as all data were deidentified by the faculty before distribution to researchers. The HF diagnosis was based on specific ICD-10 codes (N = 31,278). To ensure accuracy, we excluded patients not using diuretics (N = 6,081), individuals under 20 years old (N = 14), and those without complete echocardiography data needed for HF classification (N = 16,108) (Supplementary Figure 1).

### Patients inclusion

Patients were included in the study cohort based on a clinical diagnosis of HF, an EF of 40%–49%, and a condition duration of at least 3 months to allow for the optimization of treatment. The study targeted patients with HFmrEF as defined by the guidelines, identifying them as the primary study population. Patients with HF with reduced EF (HFrEF), characterized by an EF of less than 40%, were incorporated as a positive control group due to the well-documented beneficial outcomes associated with RASI/ARNI and MRA in this subgroup (see Supplementary Table 1). The exposure of primary interest was use of MRA and use of RASI/ARNI, while the non-user group was also likely to receive other heart failure medication treatment.

### Outcomes definition

The primary outcome measured was a composite of cardiovascular (CV) mortality and HHF. Secondary outcomes included the individual metrics of CV mortality and HHF. HHF was defined as an unscheduled hospitalization during which the patient required at least one treatment, such as diuretics, nitrites, or inotropic agents. Data on the date and cause of death were accessible by linking to the Taiwan Death Registry database. Additionally, a falsification (negative control) analysis was conducted, examining the link between medication use and hospitalizations for psoriasis and influenza in HFmrEF patients. This analysis aims to identify any potential residual confounding by testing an association that is theoretically non-existent; a positive correlation would suggest the presence of such confounding (Lipsitch et al., 2010).

### Statistical analysis

The baseline characteristics of patients with and without drug use (i.e., MRA and RASI/ARNI) were presented as follows: frequency and percentage for categorical variables, mean and standard deviation for continuous variables, and median and interquartile range for the apparently skewed continuous variable (i.e., NT-Pro BNP). To balance the baseline characteristics between the study groups (e.g., MRA vs. non-MRA), we established inverse probability treatment weighting (IPTW) adjusted cohorts based on propensity score when comparing outcomes. The propensity scores were computed using all covariates (listed in Table 1), except for replacing the follow-up year with the index date. The propensity scores were estimated using generalized boosted model with 10,000 trees and an estimand of average treatment effect (McCaffrey et al., 2013). The balance of baseline characteristics between groups was evaluated using the standardized difference (STD), with an absolute STD value less than 0.2 considered indicative of a non-substantial group difference (McCaffrey et al., 2013). Furthermore, because a significant amount of laboratory data was missing, the data underwent imputation using a single expectation maximization algorithm prior to further analysis.

**TABLE 1:** Baseline characteristics of patients with HFmrEF according to use of mineralocorticoid receptor antagonists or renin-angiotensin system inhibitor/angiotensin receptor neprilysin inhibitor in the original cohort.

Variable	Available Number	Total (*n* = 2,584)	MRA			RASI/ARNI		
			User (*n* = 444)	Non-user (*n* = 2,140)	STD	User (*n* = 1,101)	Non-user (*n* = 1,483)	STD
Demographics								
Age, year	2,584	67.5 ± 15.2	65.2 ± 16.0	68.0 ± 14.9	−0.18	67.5 ± 14.4	67.5 ± 15.7	<0.01
Male sex	2,584	1,706 (66.0)	303 (68.2)	1,403 (65.6)	0.06	765 (69.5)	941 (63.5)	0.13
Body mass index, kg/m^2^	2,443	24.6 ± 5.7	24.7 ± 4.5	24.6 ± 5.9	0.03	24.8 ± 4.5	24.5 ± 6.4	0.08
Smoking	2,584	122 (4.7)	32 (7.2)	90 (4.2)	0.14	58 (5.3)	64 (4.3)	0.04
LVEF, %	2,584	44.9 ± 2.8	44.6 ± 2.9	44.9 ± 2.8	0.12	44.9 ± 2.8	44.9 ± 2.8	−0.01
Comorbidity								
Hypertension	2,584	940 (36.4)	179 (40.3)	761 (35.6)	0.10	592 (53.8)	348 (23.5)	0.63
Diabetes mellitus	2,584	676 (26.2)	118 (26.6)	558 (26.1)	0.01	407 (37.0)	269 (18.1)	0.43
Dyslipidemia	2,584	545 (21.1)	114 (25.7)	431 (20.1)	0.14	370 (33.6)	175 (11.8)	0.53
Coronary artery disease	2,584	1,031 (39.9)	181 (40.8)	850 (39.7)	0.02	579 (52.6)	452 (30.5)	0.45
Myocardial infarction	2,584	348 (13.5)	61 (13.7)	287 (13.4)	0.01	205 (18.6)	143 (9.6)	0.26
Atrial fibrillation	2,584	343 (13.3)	103 (23.2)	240 (11.2)	0.35	168 (15.3)	175 (11.8)	0.10
COPD	2,584	74 (2.9)	16 (3.6)	58 (2.7)	0.05	36 (3.3)	38 (2.6)	0.04
Ischemic stroke	2,584	297 (11.5)	55 (12.4)	242 (11.3)	0.03	151 (13.7)	146 (9.8)	0.12
Chronic kidney disease	2,584	842 (32.6)	98 (22.1)	744 (34.8)	−0.27	317 (28.8)	525 (35.4)	−0.14
ESRD with dialysis	2,584	185 (7.2)	13 (2.9)	172 (8.0)	−0.20	81 (7.4)	104 (7.0)	0.01
Laboratory data								
NT-Pro BNP, pg/mL	1,150	4,245 [1760, 10,100]	3,390 [1,418, 7,660]	4,435 [1816, 10,636]	−0.25	3,756 [1,402, 8,326]	4,642 [1995, 10,899]	−0.17
≥Median		575 (50.0)	89 (39.7)	486 (52.5)		227 (45.2)	348 (53.7)	
<Median		575 (50.0)	135 (60.3)	440 (47.5)		275 (54.8)	300 (46.3)	
Missing	1,434							
Uric acid, mg/dL	1,002	7.2 ± 2.4	7.4 ± 2.6	7.1 ± 2.4	0.07	7.0 ± 2.2	7.4 ± 2.6	−0.09
Hyperuricemia (≥7)		487 (48.6)	108 (52.9)	379 (47.5)		244 (44.9)	243 (53.1)	
Normal (<7)		515 (51.4)	96 (47.1)	419 (52.5)		300 (55.2)	215 (46.9)	
Missing	1,582							
Potassium (K), mEq/L	2,130	4.2 ± 0.6	4.1 ± 0.6	4.2 ± 0.6	−0.04	4.2 ± 0.6	4.1 ± 0.7	0.06
Hyperkalemia (≥5)		183 (8.6)	31 (8.6)	152 (8.6)		67 (7.4)	116 (9.4)	
Normal (3.5–5)		1,686 (79.2)	282 (78.1)	1,404 (79.4)		757 (84.0)	929 (75.6)	
Hypokalemia (≤3.5)		261 (12.3)	48 (13.3)	213 (12.0)		77 (8.6)	184 (15.0)	
Missing	454							
eGFR, mL/min/1.73m^2^	2,284	63.1 ± 38.9	71.8 ± 34.5	61.3 ± 39.5	0.26	64.2 ± 36.2	62.2 ± 40.9	0.05
<30		529 (23.2)	44 (11.3)	485 (25.6)		196 (19.8)	333 (25.8)	
30–60		569 (24.9)	103 (26.6)	466 (24.6)		242 (24.4)	327 (25.3)	
≥60		1,186 (51.9)	241 (62.1)	945 (49.8)		553 (55.8)	633 (49.0)	
Missing	300							
Medication								
MRA	2,584	444 (17.2)	444 (100)	0 (0)	—	250 (22.7)	194 (13.1)	0.26
RASI/ARNI	2,584	1,101 (42.6)	250 (56.3)	851 (39.8)		1,101 (100)	0 (0)	—
Beta-blocker	2,584	932 (36.1)	225 (50.7)	707 (33.0)	0.37	627 (57.0)	305 (20.6)	0.76
SGLT2i	2,584	42 (1.6)	14 (3.2)	28 (1.3)	0.15	32 (2.9)	10 (0.7)	0.18
Ivabradine	2,584	10 (0.4)	7 (1.6)	3 (0.1)	0.23	5 (0.5)	5 (0.3)	0.02
Antiplatelet	2,584	383 (14.8)	107 (24.1)	276 (12.9)	0.01	186 (16.9)	197 (13.3)	0.67
CCB	2,584	782 (30.3)	123 (27.7)	659 (30.8)	−0.07	446 (40.5)	336 (22.7)	0.39
OHA^a^	2,584	500 (19.4)	93 (21.0)	407 (19.0)	0.08	312 (28.3)	188 (12.7)	0.44
Insulin	2,584	502 (19.4)	82 (18.5)	420 (19.6)	−0.03	232 (21.1)	270 (18.2)	0.07
GLP1-RA	2,584	3 (0.1)	1 (0.2)	2 (0.1)	0.04	2 (0.2)	1 (0.1)	0.03
DOAC	2,584	111 (4.3)	35 (7.9)	76 (3.6)	0.21	63 (5.7)	48 (3.2)	0.12
Follow up duration, year	2,584	3.5 ± 3.3	3.6 ± 3.3	3.4 ± 3.3	−0.04	4.0 ± 3.5	3.1 ± 3.1	−0.26

Abbreviation: ARNI, angiotensin receptor neprilysin inhibitor; HFmrEF, heart failure with mid-range ejection fraction; MRA, mineralocorticoid receptor antagonists; RASI, renin-angiotensin system inhibitor; STD, standardized difference; LVEF, left ventricular ejection fraction; COPD, chronic obstructive pulmonary disease; ESRD, end stage renal disease; NT-Pro BNP, N-terminal pro-brain natriuretic peptide; eGFR, estimated glomerular filtration rate; SGLT2i, sodium-glucose cotransporter 2 inhibitors; CCB, calcium channel blockers; OHA, oral hypoglycemic agent; GLP1-RA, glucagon-like peptide-1, receptor agonist; DOAC, direct oral anticoagulants.

^a^ Not including SGLT2i; Data are presented as frequency (percentage), mean ± standard deviation or median [25th, 75th percentiles].

We employed a series of univariate logistic regression models to explore potential predictors of treatment utilization. The association between treatment usage and the risk of fatal outcomes (i.e., primary outcome, CV death and non-CV death) was tested using the Cox proportional hazards model. The incidence of non-fatal outcomes between the study groups was compared using Fine and Gray subdistribution hazard model, which accounted for all-cause death as a competing risk. Moreover, guided on our findings, we conducted a landmark analysis on the primary outcome at the initial 2 years of the observation period. The IPTW was conducted with R version 4.3.2 (R Project for Statistical Computing) and the package “twang.” The remaining analyses were performed using SAS version 9.4 (SAS Institute, Cary, NC).

The data that support the findings of this study are available from the corresponding author, provided that data sharing is permitted by National Taiwan University Hospital appropriate ethics committees.

## Results

Of 2,584 patients with HFmrEF, 1,101 (42.6%) received RASI and 444 (17.2%) received MRA. Patients treated with both RASI/ARNI and MRA were 250 (9.7%), 1,045 (40.4%) received one drug, and 1,289 (49.9%) received neither drug. Mean age was 67.5 ± 15.2 years and 66% were males (Table 1).

### Baseline characteristics and predictors of treatments

#### MRA

Table 1 shows baseline characteristics of patients based on MRA usage. Among 2,584 patients, 444 used MRAs. MRA users were younger (65.2 vs. 68.0 years) and more likely to be male (68.2% vs. 65.6%). Atrial fibrillation was more common in MRA users (23.2% vs. 11.2%), suggesting a possible association. MRA users had lower rates of chronic kidney disease (22.1% vs. 34.8%) and end-stage renal disease dialysis (2.9% vs. 8.0%). Median NT-Pro BNP levels were lower in MRA users (3,390 vs. 4,435 pg/mL). Potassium levels showed no significant difference. A higher proportion of MRA users were on beta-blockers (50.7%), loop diuretics (66.9%), and antiplatelet therapy (24.1%).

**FIGURE 1:**
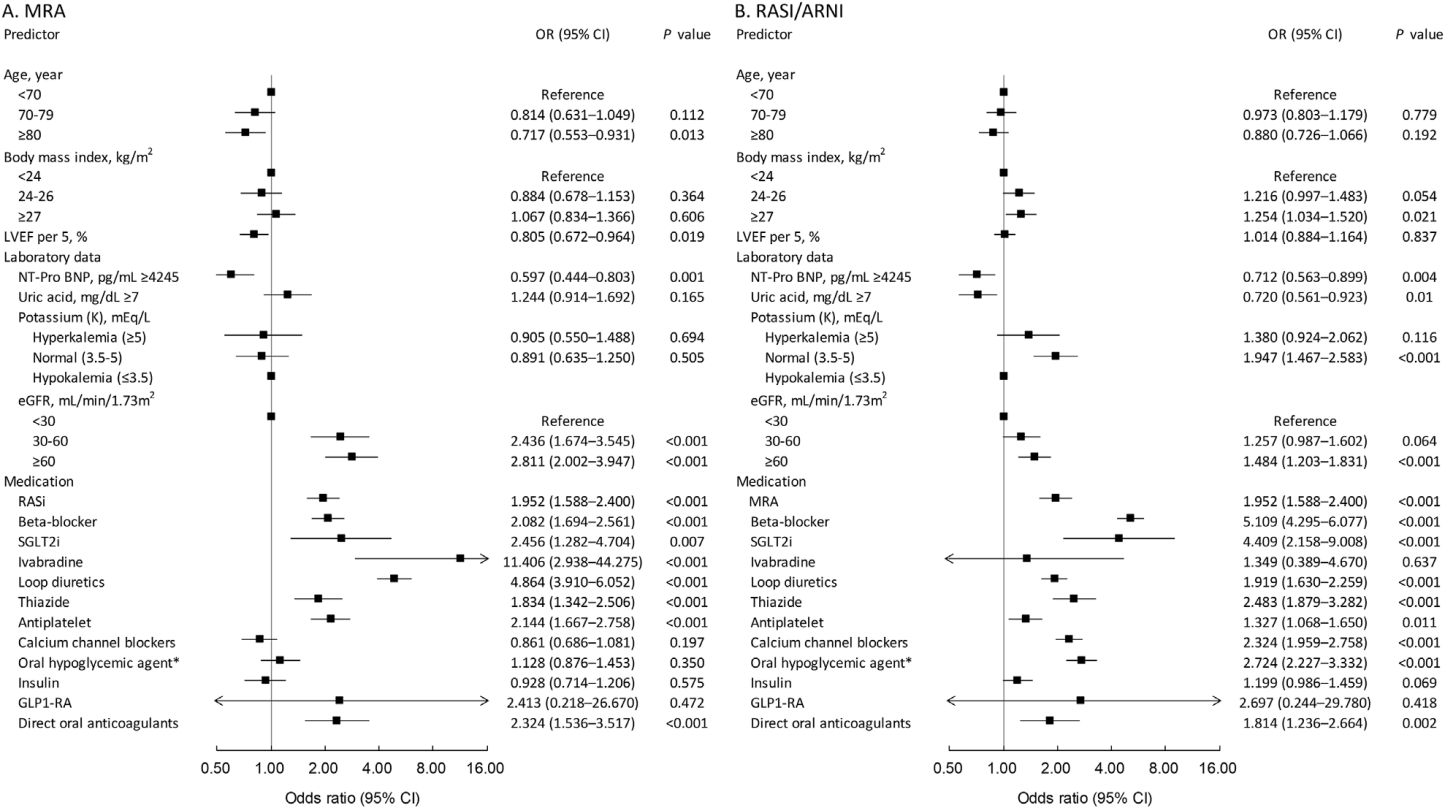
Predictors of treatment with MRA **(A)** and RASi **(B)** in the HFmrEF cohort.

Figure 1A identifies predictors of MRA use. Advanced age, particularly 80+, was inversely related to MRA use (odds ratio = 0.717, *p* = 0.013). Higher LVEF reduced MRA usage (odds ratio = 0.805 per 5% increase, *p* = 0.019). Lower renal function (eGFR < 60) increased MRA use (odds ratio = 2.436 for eGFR < 30, *p* < 0.001; odds ratio = 2.811 for eGFR 30–60, *p* < 0.001). Higher NT-pro BNP (>4,245 pg/mL) was linked to less MRA use (odds ratio = 0.597, *p* = 0.003). Potassium levels did not significantly affect MRA use. Use of RAS inhibitors, beta-blockers, SGLT2 inhibitors, ivabradine, loop and thiazide diuretics, antiplatelet medications, and direct oral anticoagulants predicted MRA use.

#### RASI/ARNI

Table 1 shows that 1,101 patients (42.6% of the study population) used RASI/ARNI, with an average age of 67.5 years, similar to non-users. The user group consisted of more males (69.5% vs. 63.5%). RASI/ARNI users had higher prevalence of hypertension (53.8%), diabetes mellitus (37.0%), dyslipidemia (33.6%), coronary artery disease (52.6%), and history of myocardial infarction (18.6%) compared to non-users. There was also a slightly higher incidence of atrial fibrillation (15.3% vs. 11.8%). Median NT-Pro BNP levels were lower in users (3,756 pg/mL vs. 4,642 pg/mL), and renal function was better (average eGFR of 64.2 mL/min/1.73 m^2^). In addition, RASI/ARNI users were prescribed with beta-blockers, loop diuretics, and calcium channel blockers more frequently.

Figure 1B identifies predictors for RASI/ARNI use. Patients with a BMI of 27 or higher were more likely to use RASI/ARNI (odds ratio = 1.254, *p* = 0.021). Age was not a significant factor. Higher NT-pro-BNP levels (>4,245 pg/mL) reduced RASI/ARNI use (odds ratio = 0.712, *p* = 0.004). Higher uric acid levels (>7 mg/dL) also decreased use (odds ratio = 0.720, *p* = 0.01). Normal potassium levels were associated with increased use (odds ratio = 1.947, *p* < 0.001). Better renal function (eGFR > 60 mL/min/1.73 m^2^) correlated with higher RASI/ARNI use (odds ratio = 1.484, *p* < 0.001). Concurrent use of MRAs, beta-blockers, and loop diuretics strongly predicted RASI/ARNI use, indicating integrated treatment for severe heart failure.

After bias correction with IPTW, there were no significant differences in baseline comorbidities and laboratory results between users and non-users, indicating balanced cohorts (Supplementary Table 1).

### Outcome analysis

Table 2 summarizes follow-up outcomes for HFmrEF patients treated with MRA or RASI/ARNI, adjusted using IPTW. For MRA-treated patients, the primary outcome rate (HHF plus cardiovascular death) was 33.0 per 100 person-years versus 36.6 in non-MRA users, with no significant risk difference (hazard ratio = 0.96, *p* = 0.22). Cardiovascular mortality was slightly lower in the MRA group (4.7 vs. 5.3 events per 100 person-years), approaching significance (hazard ratio = 0.89, *p* = 0.10), while HHF rates were similar between groups.

**TABLE 2:** Follow up outcome of patients with HFmrEF according to use of mineralocorticoid receptor antagonists or renin-angiotensin system inhibitor in the IPTW-adjusted cohort.

Exposure/Outcome	User		Non-user		HR/SHR (95% CI)	*p* value
	Event rate (%)	Incidence (95% CI)^a^	Event rate (%)	Incidence (95% CI)^a^		
MRA						
Efficacy outcome						
Primary outcome^b^	71.4	33.0 (31.3–34.7)	64.3	36.6 (34.8–38.4)	0.96 (0.89–1.03)	0.22
Cardiovascular death	17.4	4.7 (4.2–5.2)	18.4	5.3 (4.8–5.8)	0.89 (0.78–1.02)	0.10
HHF	62.4	28.9 (27.3–30.5)	56.5	32.1 (30.5–33.8)	1.01 (0.94–1.09)	0.80
Falsification endpoint						
Psoriasis	0.42	0.11 (0.04–0.19)	0.27	0.08 (0.02–0.14)	1.54 (0.56–4.19)	0.40
Influenza	0.52	0.14 (0.06–0.23)	0.85	0.25 (0.14–0.35)	0.61 (0.29–1.25)	0.17
RASI/ARNI						
Efficacy outcome						
Primary outcome^b^	68.8	32.5 (30.9–34.2)	63.9	38.7 (36.8–40.7)	0.91 (0.85–0.98)	0.01
Cardiovascular death	17.4	4.4 (3.9–4.8)	18.3	5.6 (5.1–6.1)	0.82 (0.71–0.94)	0.004
HHF	60.2	28.5 (27.0–30.0)	56.5	34.3 (32.4–36.1)	0.995 (0.924–1.070)	0.88
Falsification endpoint						
Psoriasis	0.63	0.16 (0.08–0.24)	0.15	0.05 (0.00–0.10)	4.02 (0.87–12.69)	0.18
Influenza	0.95	0.24 (0.14–0.34)	0.53	0.16 (0.07–0.25)	1.71 (0.86–3.39)	0.12

Abbreviation: ARNI, angiotensin receptor neprilysin inhibitor; IPTW, inverse probability treatment weighting; HFmrEF, heart failure with mid-range ejection fraction; CI, confidence interval; HR, hazard ratio; SHR, subdistribution hazard ratio; MRA, mineralocorticoid receptor antagonists; HHF, hospitalization for heart failure; CV, cardiovascular; RASi, renin-angiotensin system inhibitor.

^a^ Number of events per 100 person-years.

^b^ Composite of HHF, and cardiovascular death.

For RASI/ARNI-treated patients, the primary outcome rate was significantly lower (32.5 vs. 38.7 events per 100 person-years, hazard ratio = 0.91, *p* = 0.01). Cardiovascular deaths were also less frequent among RASI/ARNI users (4.4 vs. 5.6 events, hazard ratio = 0.82, *p* = 0.004), with no significant difference in HHF rates between users and non-users. In regard to the effect between RASI and ARNI (Supplementary Table 2), there was no significant difference regarding the primary outcome rate (33.3 vs. 33.0 events per 100 person-years, hazard ratio = 1.00, *p* = 0.98).

We have further investigated the effect of each treatment groups (MRA + RASI/ARNI, RASI/ARNI alone, MRA alone and non-users) (Supplementary Table 3). Taking the group of MRA + RASI/ARNI as reference, the primary outcome rate was higher in the group of MRA alone (39.9 events per 100 person-years, hazard ratio = 1.16, *p* < 0.001) and non-users (36.6 events per 100 person-years, hazard ratio = 1.08, *p* = 0.04). There was no difference in the group of RASI/ARNI alone (31.9 events per 100 person-years, hazard ratio = 0.99, *p* = 0.92).

**FIGURE 2:**
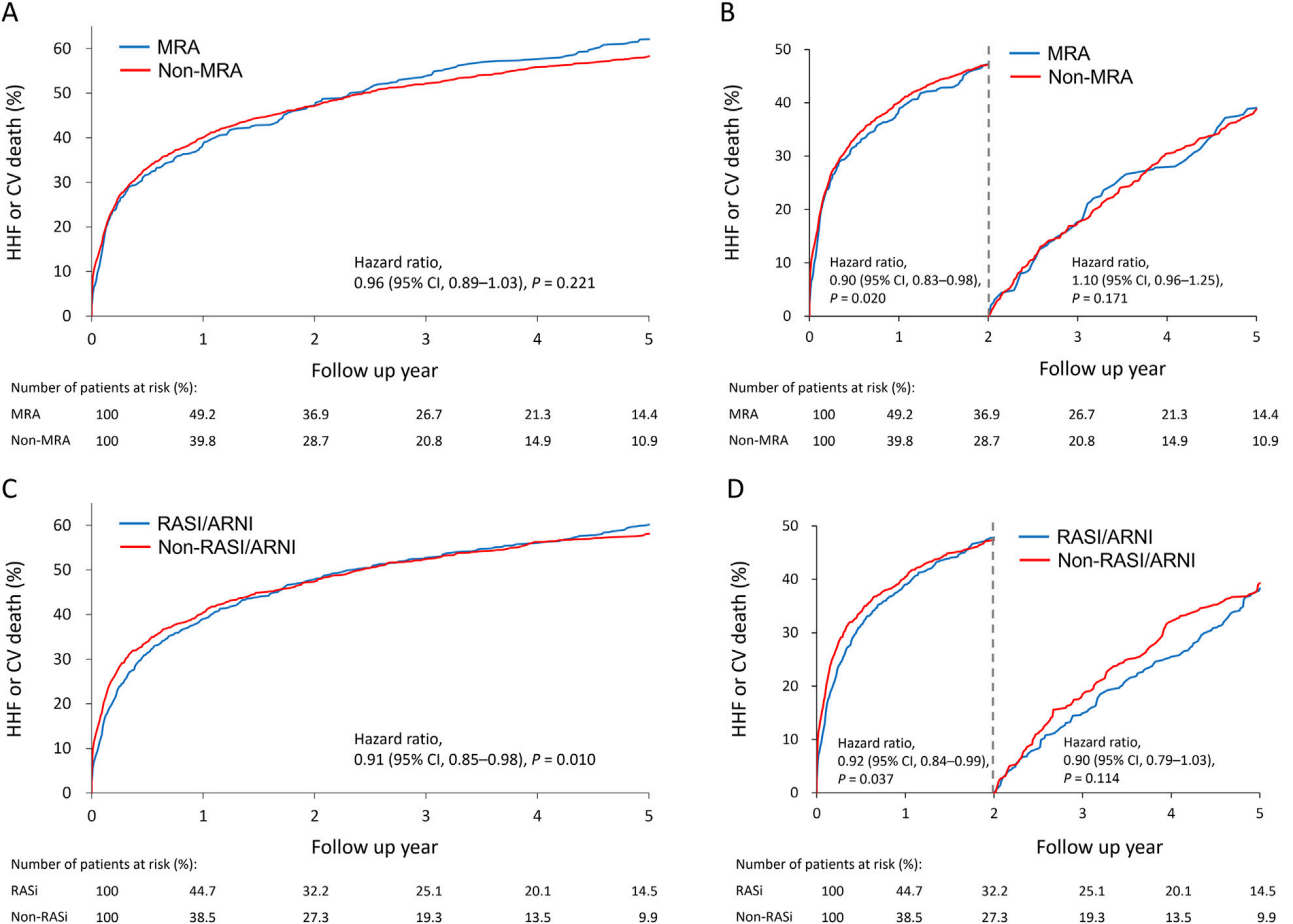
Cumulative incidence plots for the association between MRA use **(A)** and RASi use **(C)** and the primary outcome (cardiovascular death or heart failurehospitalization) and stratified Q19 by a 2-year landmark for MRA use **(B)** and RASi use **(D)** in the HFmrEF population.

**FIGURE 3:**
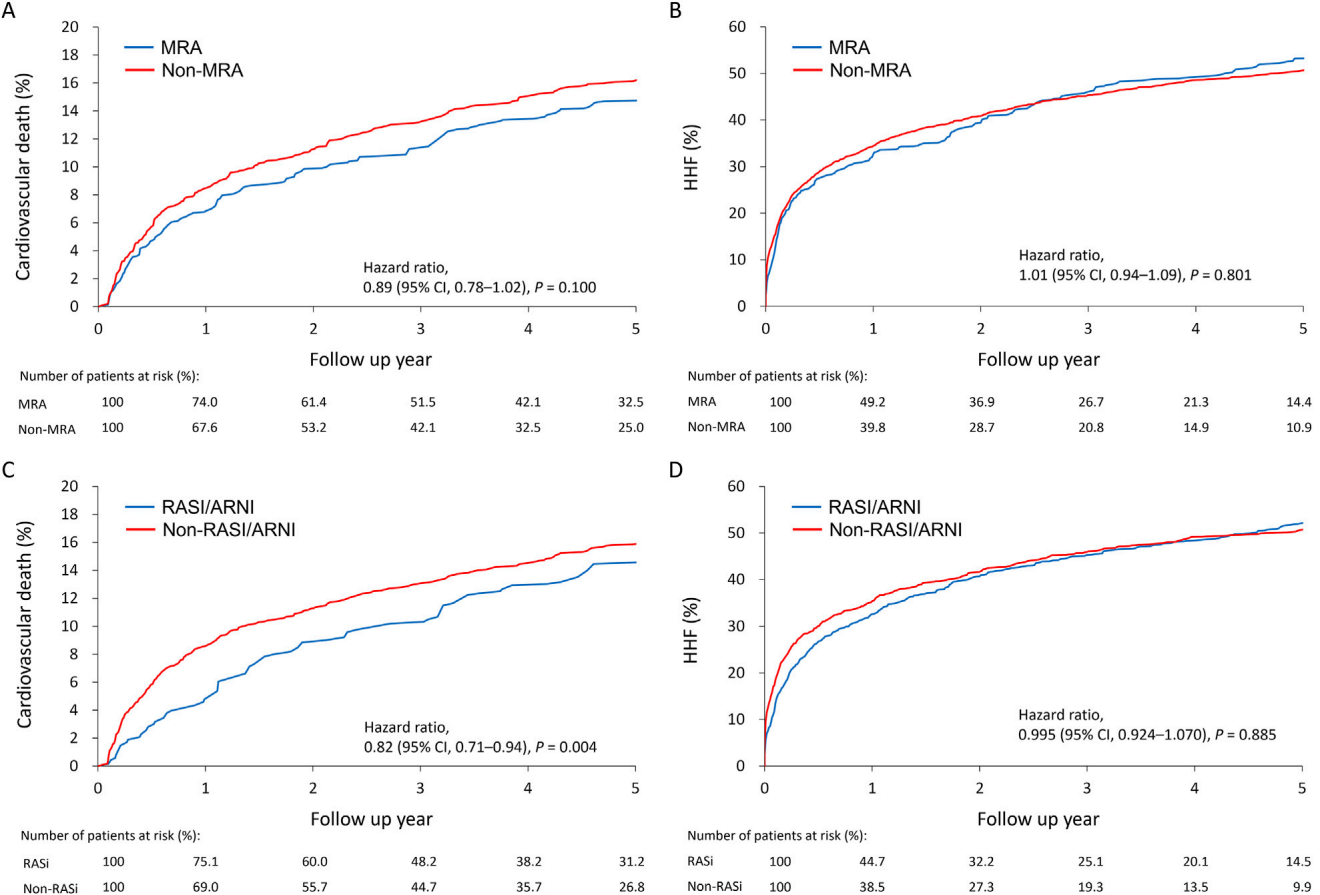
Cumulative incidence plots for the association between MRA use and the cardiovascular death **(A)** or heart failure hospitalization **(B)** and between RASI/ARNI use and the cardiovascular death **(C)** or heart failure hospitalization **(D)** in the HFmrEF population.

Cumulative incidence plots showed no significant difference in the combined risk of HHF or cardiovascular death between MRA users and non-users (Figure 2A). A 2-year landmark analysis showed a temporary risk reduction for MRA users (hazard ratio = 0.90, *p* = 0.020), which faded with longer follow-up (Figure 2B). Overall, the primary outcome incidence was lower among RASI/ARNI users (hazard ratio = 0.91, *p* = 0.01) (Figure 2C). The benefit of RASI/ARNI was evident in the first 2 years (hazard ratio = 0.92, *p* = 0.037) but tended to be non-significant afterward (Figure 2D). Kaplan-Meier curves showed similar trends in cardiovascular death and HHF rates between MRA users and non-users (Figures 3A, B). For RASI/ARNI users, there was a significant reduction in cardiovascular mortality (Figure 3C), while HHF rates remained similar between groups (Figure 3D). These results suggest that RASI/ARNI reduces cardiovascular mortality but has a less pronounced effect on HHF rates.

The figure indicates a dynamic shift in medication usage over 2 years, with MRA use increasing from 41.52% to 53.37% and RASI/ARNI use rising from 15.94% to 45.98%. SGLT2 inhibitor adoption also surged, from 1.62% to 23.25% within the MRA group and from 1.63% to 16.10% within the RASI/ARNI group, reflecting growing recognition of the benefits of SGLT2i therapy alongside MRA and RASI/ARNI treatments.

### Negative control analysis

Psoriasis and influenza were used as falsification endpoints to test the robustness of the findings (Table 2). For MRA users, psoriasis incidence was 0.11 events versus 0.08 for non-users (hazard ratio = 1.54, *p* = 0.402). Influenza incidence was 0.14 events for users versus 0.25 for non-users (hazard ratio = 0.61, *p* = 0.176). Neither showed statistical significance. For RASI/ARNI users, psoriasis incidence was 0.16 events versus 0.05 for non-users (hazard ratio = 4.02, *p* = 0.18), and influenza incidence was 0.24 events versus 0.16 for non-users (hazard ratio = 1.71, *p* = 0.128).

### Positive control analysis

Baseline characteristics of the positive control population with HFrEF (EF < 40%) are detailed in Supplementary Table 4; 21.9% were treated with MRA and 36.6% with RASI/ARNI. The IPTW-adjusted cohort is in Supplementary Table 5. After IPTW adjustment, MRA users had a 77.1% event rate of primary outcomes compared to 72.1% for non-users (Supplementary Table 6), with incidence rates of 43.3 vs. 46.1 events per 100 person-years (hazard ratio = 0.94, *p* = 0.014), indicating a significant risk reduction for MRA users. RASI/ARNI users had a 72.5% event rate compared to 73.3% for non-users, with incidence rates of 40.8 vs. 49.3 per 100 person-years (hazard ratio = 0.86, *p* < 0.001), showing a significant risk reduction for RASI/ARNI users. For both MRA and RASI/ARNI, the falsification endpoints (psoriasis and influenza) showed no significant differences, suggesting the observed effects on primary outcomes are due to the treatments rather than confounding factors. Cumulative incidence plots are illustrated in Supplementary Figure 2A, B.

## Discussions

In this extensive cohort study, 17.2% of patients with HFmrEF were treated with MRA, and 42.6% received RASI/ARNI. The prescription rates of these medications aligns with findings from previous studies conducted in the same demographic (Tsuji et al., 2017a; Lund et al., 2018; Savarese et al., 2022). To address baseline imbalances, we employed IPTW to assess outcomes in the HFmrEF cohort. The use of RASI/ARNI was linked to a significantly lower risk of primary outcomes, including a notable reduction in the risk of cardiovascular death. Importantly, this protective effect was especially pronounced during the first 2 years.

### Use and predictors of treatment use in HFmrEF

In our heart failure (HF) cohort with complete echocardiography data, we identified those patients using diuretics, indicating a minimum of NYHA Functional Class II. Out of 7,461 subjects in our cohhort, 2,584 (34%) individuals were diagnosed as HFmrEF. The proportion of HFmrEF in our HF cohort was higher than those in ESC-HF-LT registry (24%) (Koh et al., 2017), CHARM programme (17%) (Lund et al., 2018) and an Asian cohort study (Lam et al., 2018). The most often comorbidities in our HFmrEF cohort were hypertension (36.4%), CAD/MI (53.4%) and CKD/ESRD (40%). The incidences of hypertension and CAD/MI align with previously published data, whereas CKD/ESRD’s prevalence has been less explored, particularly in the Asian context (Koh et al., 2017; Tsuji et al., 2017b; Lam et al., 2018; Lund et al., 2018; Stolfo et al., 2023). Notably, renal dysfunction significantly influences the prescription of RASI/ARNI and MRA in clinical settings. The factors contributing to the prescription of both medications were associated with an eGFR higher than 60 mL/min/1.73m^2, potentially due to the enhanced safety profile of these drugs in patients with normal kidney function, particularly with RASI/ARNI (Greene et al., 2018; Stolfo et al., 2023). There was a notable increase in the prescription of RASI/ARNI among patients with normal potassium levels, indicating concerns about potential hyperkalemia caused by treatment or a reciprocal relationship, leading to elevated potassium levels in patients taking RASI/ARNI (Greene et al., 2018; Stolfo et al., 2023). Interestingly, potassium levels did not significantly influence the use of MRA, which could be attributed to the lower incidence of MRA-induced hyperkalemia in the Asian population compared to the use of RASI/ARNI in treating heart failure (Kashihara et al., 2019). Conversely, a reduced NT-proBNP level was linked to a higher utilization of RASI/ARNI and MRA, potentially due to a reverse causation effect, resulting in elevated NT-proBNP levels in patients exhibiting poor functional status. Furthermore, the simultaneous administration of drugs like beta-blockers, sodium-glucose co-transporter 2 inhibitors, loop diuretics, thiazides, antiplatelet agents, and oral anticoagulants correlated with a heightened prescription of both RASI/ARNI and MRA. This indicates a diminished risk of hypotension and a more controlled and improved heart failure condition (Gjyriqi et al., 2023). Distinct factors leading to the prescription of RASI/ARNI were a higher BMI and normal levels of uric acid, indicating that hypertension associated with a higher BMI encouraged the use of RASI/ARNI and a decreased use of diuretics when uric acid levels were normal (Choi et al., 2005). On the other hand, our HFmrEF cohort showed a higher usage rate of MRA (40%) compared to that reported in two Asian studies (11%–32%) (Tsuji et al., 2017a; Lam et al., 2018). Being over 80 years old and having an increased ejection fraction were identified as factors influencing the use of MRA. MRA has been shown to decrease morbidity and mortality among elderly heart failure patients, with this positive impact being more pronounced in those with HFrEF, although it is consistent across both HFrEF and HFpEF categories (Ferreira et al., 2019). Moreover, the extensive use of diuretics, which can lead to hypokalemia, may promote the use of MRA to help maintain potassium levels.

### Association of treatment and outcomes

In our study, RASI/ARNI had clinical benefit regarding CV mortality, and the result was similar with other studies (Lund et al., 2018; Solomon et al., 2019; Stolfo et al., 2023). In the CHARM study, HFmrEF treated with candesartan had a 24% reduction of CV death or HHF, and a 52% reduction of recurrent hospitalization (Lund et al., 2018). In the PARAGON-HF trial, though there was no overall statistically significant effect, subgroup analysis of patients with EF 45%–57% treated with ARNI did have a 16% reduction of CV death or HHF compared with those treated with Valsartan (Solomon et al., 2019). In our study, subgroup analysis for patients treated with ARNI did not have a significant clinical benefit. The reason was likely due to small case numbers. The total patients on ARNI only consisted of 3.3% of all the patients receiving RASI/ARNI.

In another meta-analysis reviewing 16 studies and 1937 patients mainly in China, treatment with RASI led to improving LV function and reducing rehospitalization rate (Qin et al., 2022). The mechanism behind was also thought to be the inhibition of neurohormonal overactivation, similar with that behind HFrEF patients (Consensus Trial Study Group, 1987; Mann and Felker, 2021; Perrone-Filardi et al., 2022).

As for MRA, the clinical benefit was neutral, even when the components of the primary outcome were separately analyzed. The result was consistent with that of the TOPCAT trial, which enrolled 520 patients with EF 45%–50% and showed a potential positive efficacy without statistically significancy (Solomon et al., 2016b). Interestingly, the clinical benefit shown in the previous studies was mainly about reduction of HF hospitalization, not about CV death (Lund et al., 2018; Solomon et al., 2019). However, in our study, we found a significant survival benefit when using RASI/ARNI. There were several possible reasons. First, our study focused on HFmrEF, and was not a sub-group analysis. Second, the basic NT-ProBNP level in our study was 4,245 pg/mL, comparing with 904 pg/mL in PARAGON-HF trial, indicating higher severity. Finally, the median following duration was 3.5 years, which was much longer than other studies. The duration in the studies above was all less than 2 years (Vaduganathan et al., 2023).

In the subgroup analysis of each treatment group including MRA + RASI/ARNI, RASI/ARNI alone, MRA alone and non-users, the clinical benefit was only observed in the group which contained RASI/ARNI, including MRA + RASI/ARNI and RASI/ARNI alone. The result suggested that there might be no synergic effect.

### Neutral protective effect of RASI/ARNI in the long-term follow-up

Another interesting finding in our study was the potential therapeutic resistance of RASI/ARNI. Though the result showed an overall therapeutic benefit with RASI/ARNI, the association became non-significant after 2 years in the landmark analysis. The median follow-up duration of our study is 3.5 years, which was longer than previous studies. The following duration of CHARM programme was 2 years (Pfeffer et al., 2003). The duration of PARAGON-HF trial was 2.5 years (Solomon et al., 2019). The longest following duration in the Chinese meta-analysis was 2 years (Qin et al., 2022). As time goes on, EF tends to change owing to effects of therapy or the natural progression of HF, either improving or deteriorating (Lam and Teng, 2016; Lupón et al., 2019; Savarese et al., 2019). In a retrospective analysis of 4,942 patients in SwedeHF, 37% and 25% of patients with HFmrEF switched to HFrEF and HFpEF, respectively; whereas 16% and 21% of patients with HFrEF or HFpEF switched to HFmrEF (Savarese et al., 2022). Several factors were associated with increasing EF (female sex, atrial fibrillation and less severe HF) or decreasing EF (diabetes, IHD and more severe HF) (Savarese et al., 2019). Patients with recovered EF had better outcomes in terms of cardiovascular death and hospitalization for HF in either the HFpEF or the HFrEF subgroup (Kalogeropoulos et al., 2016; Lupón et al., 2017). The observed variation in the change of EF could potentially result in distinct clinical outcomes, suggesting the need for tailored treatment approaches. Medication usage was initially determined at the baseline; however, subsequent changes in treatment during the follow-up period might have obscured the relationship with clinical outcomes. The data demonstrate a substantial rise in RASI/ARNI usage from 15% to 45% over 2 years, indicating that many individuals who were not on these treatments initially had started using them within this timeframe. This shift could account for the lack of observed difference in outcomes between baseline-defined users and non-users at the 2-year landmark analysis. Moreover, the notable increase in SGLT2 inhibitor use within the same period could potentially mask the beneficial effects of RASI/ARNI, particularly concerning cardiovascular mortality (Wang et al., 2022; McDonagh et al., 2023a).

### Limitations

Given the retrospective nature of this study, the potential influence of unmeasured or unknown confounders persists despite comprehensive adjustments using inverse probability of treatment weighting (IPTW). The low percentage of patients not treated with RASI/ARNI and MRA resulted in a considerable decrease in sample size and consequently, statistical power after matching. Nonetheless, our findings are consistent with the primary results of other studies and remain solid after conducting both positive and negative control analyses. While the effects of beta-blockers were not specifically examined in our study—despite approximately 36% of our subjects using them—our outcome analysis factored in their use, given their common prescription in cases of HFmrEF, where the etiologies related to CAD range from 32%–91% (Savarese et al., 2022). Finally, the baseline utilization of SGLT2 inhibitors was minimal but saw a notable uptick over the subsequent 2 years. These inhibitors, however, were not part of the study parameters, as their use in treating HFmrEF was not standard clinical practice during the initial study period of 2016–2017.

## Conclusion

In our multi-center study of patients with HFmrEF, the prescription of RASI/ARNI and MRA correlated with certain demographic and clinical characteristics, existing comorbidities, and concurrent medications. RASI/ARNI treatment, in particular, was associated with improved patient outcomes, notably a reduced risk of cardiovascular death. The extent of these relationships aligns with findings from subgroup and *post hoc* analyses of randomized controlled trials that concentrate on HFmrEF populations. Notably, the initial protective effect of RASI/ARNI observed seemed to diminish and became non-significant after a 2-year follow-up period. Our data reinforce current guideline recommendations for HFmrEF management and highlight the importance of regular echocardiographic monitoring to optimize heart failure therapies over time.

## Data Availability

The raw data supporting the conclusions of this article will be made available by the authors, without undue reservation.

## Supplementary material

SUPPLEMENTAL FIGURE 1 Study flow diagram. HF, Heart failure; HFrEF, Heart failure with reduced ejection fraction; HFpEF, Heart failure with preserved ejection fraction; RASI, Renin–angiotensin system inhibitor; ARNI, Angiotensin receptor/neprilysin inhibitor; MRA, Mineralocorticoid receptor antagonist.

SUPPLEMENTAL FIGURE 2 Cumulative incidence plots for the association between MRA use **(A)** and RASI/ARNI use **(B)** and the primary outcome (cardiovascular death or heart failure hospitalization) in the HFrEF population.
