# Supplementary material for: Comparative analysis of mineralocorticoid receptor antagonists and renin-angiotensin system inhibitors/angiotensin receptor neprilysin inhibitor in heart failure with mildly reduced ejection fraction
Source: Front Pharmacol. 2024 Dec 13;15:1507326. doi: 10.3389/fphar.2024.1507326 (PMC11671259; doi:10.3389/fphar.2024.1507326)
Supplement: Supplementary file 1 [file Table1.DOCX]

**Supplemental Table 1**. Baseline characteristics of patient with HFmrEF according to use of mineralocorticoid receptor antagonists or renin-angiotensin system inhibitor in the IPTW-adjusted cohort

|  |  | MRA | | |  | RASI/ARNI | | |
| --- | --- | --- | --- | --- | --- | --- | --- | --- |
| Variable | Available  Number | User  (*n* = 2002) | Non-user  (*n* = 2544) | STD |  | User  (*n* = 2255) | Non-user  (*n* = 2416) | STD |
| Demographics |  |  |  |  |  |  |  |  |
| Age, year | 2,584 | 66.3 ± 32.5 | 67.8 ± 16.4 | -0.10 |  | 66.6 ± 21.6 | 67.7 ± 19.5 | -0.07 |
| Male sex | 2,584 | 66.1 | 65.5 | 0.01 |  | 67.3 | 65.2 | 0.04 |
| Body mass index, kg/m^2^ | 2,443 | 24.6 ± 9.2 | 24.6 ± 6.3 | <0.01 |  | 24.5 ± 6.3 | 24.6 ± 7.9 | 0.02 |
| Smoking | 2,584 | 4.7 | 4.2 | 0.02 |  | 6.1 | 4.3 | 0.09 |
| LVEF, % | 2,584 | 44.6 ± 6.1 | 44.9 ± 3.1 | 0.06 |  | 44.9 ± 4.1 | 44.9 ± 3.6 | <0.01 |
| Comorbidity |  |  |  |  |  |  |  |  |
| Hypertension | 2,584 | 37.1 | 36.4 | 0.02 |  | 41.1 | 33.0 | 0.17 |
| Diabetes mellitus | 2,584 | 22.5 | 26.2 | -0.09 |  | 29.9 | 23.5 | 0.15 |
| Dyslipidemia | 2,584 | 23.7 | 20.4 | 0.08 |  | 24.1 | 18.2 | 0.14 |
| Coronary artery disease | 2,584 | 38.9 | 39.8 | -0.02 |  | 43.8 | 38.3 | 0.11 |
| Myocardial infarction | 2,584 | 14.4 | 13.3 | 0.03 |  | 14.4 | 11.9 | 0.07 |
| Atrial fibrillation | 2,584 | 17.2 | 12.5 | 0.14 |  | 14.5 | 13.1 | 0.04 |
| COPD | 2,584 | 2.8 | 2.7 | 0.01 |  | 2.9 | 2.9 | <0.01 |
| Ischemic stroke | 2,584 | 10.6 | 11.5 | -0.03 |  | 11.3 | 12.0 | -0.02 |
| Chronic kidney disease | 2,584 | 24.9 | 33.3 | -0.18 |  | 30.3 | 34.1 | -0.08 |
| ESRD with dialysis | 2,584 | 3.8 | 7.4 | -0.14 |  | 7.4 | 7.0 | 0.02 |
| Laboratory data |  |  |  |  |  |  |  |  |
| NT-Pro BNP, pg/mL | 1,150 | 3518  [1675, 8950] | 4340  [1775, 10100] | -0.15 |  | 4120  [1590, 10100] | 4505  [1961, 10662] | -0.07 |
| ≥Median |  | 40.7 | 51.4 |  |  | 49.2 | 53.4 |  |
| <Median |  | 59.3 | 48.7 |  |  | 50.8 | 46.6 |  |
| Missing | 1,434 |  |  |  |  |  |  |  |
| Uric acid, mg/dL | 1,002 | 7.3 ± 4.8 | 7.1 ± 2.6 | 0.06 |  | 7.0 ± 3.1 | 7.2 ± 3.5 | -0.04 |
| Hyperuricemia (≥7) |  | 49.3 | 48.6 |  |  | 44.7 | 50.2 |  |
| Normal (<7) |  | 50.8 | 51.4 |  |  | 55.3 | 49.8 |  |
| Missing | 1,582 |  |  |  |  |  |  |  |
| Potassium (K), mEq/L | 2,130 | 4.2 ± 1.4 | 4.2 ± 0.7 | 0.01 |  | 4.2 ± 0.8 | 4.2 ± 0.8 | 0.02 |
| Hyperkalemia (≥5) |  | 10.7 | 8.4 |  |  | 8.0 | 9.1 |  |
| Normal (3.5-5) |  | 74.9 | 79.7 |  |  | 82.6 | 77.6 |  |
| Hypokalemia (≤3.5) |  | 14.4 | 11.9 |  |  | 9.4 | 13.3 |  |
| Missing | 454 |  |  |  |  |  |  |  |
| eGFR, mL/min/1.73m^2^ | 2,284 | 68.5 ± 75.5 | 62.6 ± 42.8 | 0.15 |  | 64.0 ± 54.1 | 62.9 ± 51.0 | 0.03 |
| <30 |  | 14.5 | 23.8 |  |  | 21.9 | 24.5 |  |
| 30-60 |  | 27.7 | 25.0 |  |  | 22.9 | 24.8 |  |
| ≥60 |  | 57.8 | 51.2 |  |  | 55.2 | 50.7 |  |
| Missing | 300 |  |  |  |  |  |  |  |
| Medication |  |  |  |  |  |  |  |  |
| MRA | 2,584 | 2002 (100) | 0 (0) | - |  | 20.7 | 16.0 | 0.12 |
| RASI/ARNI | 2,584 | 52.1 | 41.7 | 0.21 |  | 2255 (100) | 0 | - |
| Beta-blocker | 2,584 | 42.9 | 35.2 | 0.16 |  | 41.5 | 32.8 | 0.18 |
| SGLT2i | 2,584 | 2.7 | 1.4 | 0.11 |  | 2.1 | 0.9 | 0.09 |
| Ivabradine | 2,584 | 0.6 | 0.2 | 0.07 |  | 0.4 | 0.3 | 0.02 |
| Loop diuretics | 2,584 | 43.2 | 34.5 | 0.18 |  | 40.6 | 35.8 | 0.10 |
| Thiazide | 2,584 | 12.0 | 8.5 | 0.12 |  | 10.6 | 7.8 | 0.10 |
| Antiplatelet | 2,584 | 21.2 | 13.5 | <0.01 |  | 15.6 | 15.1 | 0.18 |
| CCB | 2,584 | 27.7 | 31.0 | -0.07 |  | 35.0 | 28.4 | 0.14 |
| OHA* | 2,584 | 18.0 | 19.3 | -0.01 |  | 22.1 | 17.5 | 0.15 |
| Insulin | 2,584 | 16.2 | 19.2 | -0.08 |  | 19.2 | 18.9 | 0.01 |
| GLP1-RA | 2,584 | 0.1 | 0.1 | -0.01 |  | 0.1 | 0.1 | 0.01 |
| DOAC | 2,584 | 6.5 | 3.9 | 0.13 |  | 5.2 | 3.4 | 0.09 |
| Follow up duration, year | 2,584 | 3.7 ± 6.9 | 3.5 ± 3.6 | -0.04 |  | 4.0 ± 5.0 | 3.3 ± 4.1 | -0.16 |

Abbreviation: ARNI, angiotensin receptor neprilysin inhibitor; HFmrEF, heart failure with mid-range ejection fraction; GBM, generalized boosted modeling; IPTW, inverse probability treatment weighting; MRA, mineralocorticoid receptor antagonists; RASi, renin-angiotensin system inhibitor; STD, standardized difference; LVEF, left ventricular ejection fraction; COPD, chronic obstructive pulmonary disease; ESRD, end stage renal disease; NT-Pro BNP, N-terminal pro-brain natriuretic peptide; eGFR, estimated glomerular filtration rate; SGLT2i, sodium-glucose cotransporter 2 inhibitors; CCB, calcium channel blockers; OHA, oral hypoglycemic agent; GLP1-RA, glucagon-like peptide-1 receptor agonist; DOAC, direct oral anticoagulants;

* Not including SGLT2i;

Data are presented as frequency (percentage), mean ± standard deviation or median [25^th^, 75^th^ percentiles].

**Supplemental Table 2**. Follow up outcome of patients with HFmrEF according to use of renin-angiotensin system inhibitor in the IPTW-adjusted cohort.

|  | ARNI | | ACEI/ARB | | HR/SHR | *P* |
| --- | --- | --- | --- | --- | --- | --- |
| Exposure / Outcome | Event rate (%) | Incidence (95% CI)# | Event rate (%) | Incidence (95% CI)# | (95% CI) | value |
| Efficacy outcome |  |  |  |  |  |  |
| Primary outcome* | 70.5 | 33.3 (28.7–37.9) | 69.4 | 33.0 (30.6–35.4) | 1.002 (0.858–1.170) | 0.980 |
| Cardiovascular death | 23.2 | 5.4 (4.1–6.7) | 17.5 | 4.5 (3.8–5.1) | 1.25 (0.94–1.65) | 0.121 |
| HHF | 57.0 | 27.0 (22.9–31.1) | 61.3 | 29.1 (26.9–31.4) | 0.86 (0.73–1.01) | 0.062 |
| Falsification endpoint |  |  |  |  |  |  |
| Psoriasis | 2.2 | 0.52 (0.12–0.92) | 0.3 | 0.07 (0.01–0.16) | 7.79 (0.85–29.62) | 0.093 |
| Influenza | 0.8 | 0.19 (0.05–0.43) | 1.2 | 0.30 (0.13–0.47) | 0.69 (0.17–2.84) | 0.608 |

Abbreviation: IPTW, inverse probability treatment weighting; ARNI, angiotensin receptor-neprilysin inhibitor; ACEi, angiotensin converting enzyme inhibitor; ARB, angiotensin receptor blockers; CI, confidence interval; HR, hazard ratio; SHR, subdistribution hazard ratio; HFmrEF, heart failure with mid-range ejection fraction; HHF, hospitalization for heart failure; CV, cardiovascular; HFrEF, heart failure with reduced ejection fraction;

* Composite of HHF and cardiovascular death;

# Number of events per 100 person-years.

**Supplemental Table 3**. Follow up outcome of patients with HFmrEF according to use of mineralocorticoid receptor antagonists or renin-angiotensin system inhibitor in the IPTW-adjusted cohort

| Outcome / Group | Incidence (95% CI)# | HR/SHR (95% CI) | *P* value |
| --- | --- | --- | --- |
| Primary outcome* |  |  |  |
| None | 36.6 (34.7–38.4) | 1.079 (1.004–1.160) | 0.039 |
| MRA only | 39.9 (37.8–42.0) | 1.16 (1.08–1.25) | <0.001 |
| RASI only | 31.9 (30.3–33.5) | 0.997 (0.928–1.071) | 0.927 |
| MRA + RASI | 30.3 (28.8–31.9) | Reference | - |
| Cardiovascular death |  |  |  |
| None | 5.6 (5.1–6.1) | 1.53 (1.32–1.77) | <0.001 |
| MRA only | 5.9 (5.3–6.5) | 1.58 (1.35–1.84) | <0.001 |
| RASI only | 4.4 (4.0–4.8) | 1.26 (1.08–1.47) | 0.003 |
| MRA + RASI | 3.5 (3.1–3.9) | Reference | - |
| HHF |  |  |  |
| None | 32.2 (30.4–33.9) | 1.06 (0.98–1.14) | 0.142 |
| MRA only | 35.1 (33.1–37.1) | 1.14 (1.06–1.24) | 0.001 |
| RASI only | 27.9 (26.4–29.4) | 0.97 (0.90–1.05) | 0.425 |
| MRA + RASI | 27.3 (25.8–28.8) | Reference | - |

* Composite of HHF and cardiovascular death;

# Number of events per 100 person-years.

**Supplemental Table 4**. Baseline characteristics of patient with HFrEF according to use of mineralocorticoid receptor antagonists or renin-angiotensin system inhibitor in the original cohort

|  |  |  | MRA | | |  | RASI/ARNI | | |
| --- | --- | --- | --- | --- | --- | --- | --- | --- | --- |
| Variable | Available  Number | Total  (*n* = 3,971) | User  (*n* = 873) | Non-user  (*n* = 3,098) | STD |  | User  (*n* = 1,456) | Non-user  (*n* = 2,515) | STD |
| Demographics |  |  |  |  |  |  |  |  |  |
| Age, year | 3,971 | 63.2 ± 15.7 | 61.8 ± 15.8 | 63.6 ± 15.7 | -0.11 |  | 64.1 ± 15.2 | 62.7 ± 16.0 | 0.09 |
| Male sex | 3,971 | 2,815 (70.9) | 620 (71.0) | 2,195 (70.9) | <0.01 |  | 1,041 (71.5) | 1,774 (70.5) | 0.02 |
| Body mass index, kg/m^2^ | 3,692 | 25.5 ± 17.0 | 25.3 ± 9.0 | 25.5 ± 18.7 | 0.07 |  | 25.6 ± 15.1 | 25.4 ± 18.0 | 0.07 |
| Smoking | 3,971 | 243 (6.1) | 69 (7.9) | 174 (5.6) | 0.10 |  | 100 (6.9) | 143 (5.7) | 0.05 |
| LVEF, % | 3,971 | 30.5 ± 6.6 | 30.1 ± 6.6 | 30.6 ± 6.6 | 0.06 |  | 31.0 ± 6.3 | 30.1 ± 6.7 | <0.01 |
| Comorbidity |  |  |  |  |  |  |  |  |  |
| Hypertension | 3,971 | 1,077 (27.1) | 285 (32.7) | 792 (25.6) | 0.16 |  | 641 (44.0) | 436 (17.3) | 0.6 |
| Diabetes mellitus | 3,971 | 846 (21.3) | 219 (25.1) | 627 (20.2) | 0.12 |  | 467 (32.1) | 379 (15.1) | 0.42 |
| Dyslipidemia | 3,971 | 577 (14.5) | 161 (18.4) | 416 (13.4) | 0.14 |  | 374 (25.7) | 203 (8.1) | 0.50 |
| Coronary artery disease | 3,971 | 1,238 (31.2) | 316 (36.2) | 922 (29.8) | 0.14 |  | 621 (42.7) | 617 (24.5) | 0.39 |
| Myocardial infarction | 3,971 | 394 (9.9) | 92 (10.5) | 302 (9.8) | 0.03 |  | 184 (12.6) | 210 (8.4) | 0.14 |
| Atrial fibrillation | 3,971 | 399 (10.1) | 122 (14.0) | 277 (8.9) | 0.17 |  | 179 (12.3) | 220 (8.8) | 0.12 |
| COPD | 3,971 | 73 (1.8) | 22 (2.5) | 51 (1.7) | 0.07 |  | 29 (2.0) | 44 (1.8) | 0.02 |
| Ischemic stroke | 3,971 | 321 (8.1) | 72 (8.3) | 249 (8.0) | 0.01 |  | 153 (10.5) | 168 (6.7) | 0.14 |
| Chronic kidney disease | 3,971 | 1,118 (28.2) | 172 (19.7) | 946 (30.5) | -0.24 |  | 365 (25.1) | 753 (29.9) | -0.11 |
| ESRD with dialysis | 3,971 | 211 (5.3) | 19 (2.2) | 192 (6.2) | -0.18 |  | 80 (5.5) | 131 (5.2) | 0.01 |
| Laboratory data |  |  |  |  |  |  |  |  |  |
| NT-Pro BNP, pg/mL | 1,756 | 5480  [2630, 10836] | 5363  [2380, 10678] | 5515  [2705, 10900] | -0.19 |  | 5363  [2380, 10678] | 5515  [2705, 10900] | -0.21 |
| ≥Median |  | 878 (50.0) | 190 (49.2) | 688 (50.2) |  |  | 274 (43.4) | 604 (53.7) |  |
| <Median |  | 878 (50.0) | 196 (50.8) | 682 (49.8) |  |  | 358 (56.7) | 520 (46.3) |  |
| Missing | 2,215 |  |  |  |  |  |  |  |  |
| Uric acid, mg/dL | 1,291 | 7.5 ± 2.6 | 7.8 ± 2.6 | 7.4 ± 2.5 | 0.07 |  | 7.4 ± 2.3 | 7.7 ± 2.8 | -0.16 |
| Hyperuricemia (≥7) |  | 695 (53.8) | 216 (60.2) | 479 (51.4) |  |  | 354 (53.5) | 341 (54.2) |  |
| Normal (<7) |  | 596 (46.2) | 143 (39.8) | 453 (48.6) |  |  | 308 (46.5) | 288 (45.8) |  |
| Missing | 2,680 |  |  |  |  |  |  |  |  |
| Potassium (K), mEq/L | 3,103 | 4.2 ± 0.6 | 4.1 ± 0.6 | 4.2 ± 0.6 | -0.03 |  | 4.2 ± 0.6 | 4.1 ± 0.6 | 0.07 |
| Hyperkalemia (≥5) |  | 242 (7.8) | 50 (7.5) | 192 (7.9) |  |  | 88 (7.6) | 154 (7.9) |  |
| Normal (3.5-5) |  | 2,498 (80.5) | 535 (80.0) | 1,963 (80.7) |  |  | 961 (83.4) | 1,537 (78.8) |  |
| Hypokalemia (≤3.5) |  | 363 (11.7) | 84 (12.6) | 279 (11.5) |  |  | 104 (9.0) | 259 (13.3) |  |
| Missing | 868 |  |  |  |  |  |  |  |  |
| eGFR, mL/min/1.73m^2^ | 3,336 | 65.2 ± 36.4 | 72.2 ± 32.8 | 63.3 ± 37.0 | 0.23 |  | 67.3 ± 34.3 | 63.9 ± 37.5 | 0.08 |
| <30 |  | 603 (18.1) | 68 (9.4) | 535 (20.5) |  |  | 178 (14.2) | 425 (20.4) |  |
| 30-60 |  | 883 (26.5) | 186 (25.8) | 697 (26.7) |  |  | 326 (26.0) | 557 (26.7) |  |
| ≥60 |  | 1,850 (55.5) | 467 (64.8) | 13,83 (52.9) |  |  | 748 (59.7) | 1,102 (52.9) |  |
| Missing | 635 |  |  |  |  |  |  |  |  |
| Medication |  |  |  |  |  |  |  |  |  |
| MRA | 3,971 | 873 (22.0) | 873 (100) | 0 (0 | - |  | 535 (36.7) | 338 (13.4) | 0.56 |
| RASI/ARNI | 3,971 | 1,456 (36.7) | 535 (61.3) | 921 (29.7) | 0.65 |  | 1456 (100) | 0 (0) | - |
| Beta-blocker | 3,971 | 1,153 (29.0) | 441 (50.5) | 712 (23.0) | 0.61 |  | 763 (52.4) | 390 (15.5) | 0.81 |
| SGLT2i | 3,971 | 55 (1.4) | 29 (3.3) | 26 (0.8) | 0.21 |  | 41 (2.8) | 14 (0.6) | 0.19 |
| Ivabradine | 3,971 | 34 (0.9) | 20 (2.3) | 14 (0.5) | 0.20 |  | 16 (1.1) | 18 (0.7) | 0.04 |
| Loop diuretics | 3,971 | 1,524 (38.4) | 634 (72.6) | 890 (28.7) | 0.90 |  | 828 (56.9) | 696 (27.7) | 0.60 |
| Thiazide | 3,971 | 246 (6.2) | 81 (9.3) | 165 (5.3) | 0.16 |  | 161 (11.1) | 85 (3.4) | 0.32 |
| Antiplatelet | 3,971 | 496 (12.5) | 145 (16.6) | 351 (11.3) | 0.28 |  | 232 (15.9) | 264 (10.5) | 0.61 |
| CCB | 3,971 | 765 (19.3) | 176 (20.2) | 589 (19.0) | 0.03 |  | 428 (29.4) | 337 (13.4) | 0.41 |
| OHA* | 3971 | 625 (15.7) | 168 (19.2) | 457 (14.8) | 0.16 |  | 378 (26.0) | 247 (9.8) | 0.47 |
| Insulin | 3,971 | 557 (14.0) | 119 (13.6) | 438 (14.1) | -0.01 |  | 210 (14.4) | 347 (13.8) | 0.02 |
| GLP1-RA | 3,971 | 7 (0.2) | 3 (0.3) | 4 (0.1) | 0.05 |  | 4 (0.3) | 3 (0.1) | 0.04 |
| DOAC | 3,971 | 130 (3.3) | 42 (4.8) | 88 (2.8) | 0.11 |  | 70 (4.8) | 60 (2.4) | 0.14 |
| Follow up duration, year | 3,971 | 3.5 ± 3.5 | 3.7 ± 3.5 | 3.5 ± 3.5 | -0.05 |  | 3.8 ± 3.6 | 3.4 ± 3.4 | -0.12 |

Abbreviation: ARNI, angiotensin receptor neprilysin inhibitor; HFrEF, heart failure with reduced ejection fraction; GBM, generalized boosted modeling; IPTW, inverse probability treatment weighting; MRA, mineralocorticoid receptor antagonists; RASi, renin-angiotensin system inhibitor; STD, standardized difference; LVEF, left ventricular ejection fraction; COPD, chronic obstructive pulmonary disease; ESRD, end stage renal disease; NT-Pro BNP, N-terminal pro-brain natriuretic peptide; eGFR, estimated glomerular filtration rate; SGLT2i, sodium-glucose cotransporter 2 inhibitors; CCB, calcium channel blockers; OHA, oral hypoglycemic agent; GLP1-RA, glucagon-like peptide-1 receptor agonist; DOAC, direct oral anticoagulants;

* Not including SGLT2i;

Data are presented as frequency (percentage), mean ± standard deviation or median [25^th^, 75^th^ percentiles].

**Supplemental Table 5**. Baseline characteristics of patient with HFrEF according to use of mineralocorticoid receptor antagonists or renin-angiotensin system inhibitor in the IPTW-adjusted cohort

|  |  | MRA | | |  | RASI/ARNI | | |
| --- | --- | --- | --- | --- | --- | --- | --- | --- |
| Variable | Available  Number | User  (*n* = 3263.8) | Non-user  (*n* = 3913.6) | STD |  | User  (*n* = 3084.5) | Non-user  (*n* = 3748.9) | STD |
| Demographics |  |  |  |  |  |  |  |  |
| Age, year | 3,971 | 62.8 ± 29.8 | 63.4 ± 17.6 | -0.04 |  | 63.7 ± 22.3 | 63.3 ± 19.0 | 0.02 |
| Male sex | 3,971 | 70.1 | 70.7 | -0.01 |  | 71.2 | 70.7 | 0.01 |
| Body mass index, kg/m^2^ | 3,692 | 25.5 ± 24.9 | 25.7 ± 21.5 | -0.01 |  | 25.4 ± 23.9 | 25.7 ± 25.1 | -0.02 |
| Smoking | 3,971 | 8.5 | 5.8 | 0.11 |  | 6.7 | 5.8 | 0.03 |
| LVEF, % | 3,971 | 30.0 ± 12.7 | 30.6 ± 7.4 | 0.06 |  | 30.8 ± 9.2 | 30.4 ± 8.1 | -0.05 |
| Comorbidity |  |  |  |  |  |  |  |  |
| Hypertension | 3,971 | 29.5 | 27.2 | 0.05 |  | 33.0 | 24.6 | 0.19 |
| Diabetes mellitus | 3,971 | 22.6 | 21.5 | 0.03 |  | 25.0 | 20.4 | 0.11 |
| Dyslipidemia | 3,971 | 15.2 | 14.5 | 0.02 |  | 18.3 | 13.2 | 0.15 |
| Coronary artery disease | 3,971 | 32.8 | 31.3 | 0.03 |  | 35.0 | 30.7 | 0.09 |
| Myocardial infarction | 3,971 | 10.2 | 10.0 | 0.01 |  | 10.6 | 9.6 | 0.03 |
| Atrial fibrillation | 3,971 | 11.6 | 9.5 | 0.07 |  | 10.5 | 10.5 | <0.01 |
| COPD | 3,971 | 2.6 | 1.7 | 0.07 |  | 1.6 | 1.9 | -0.02 |
| Ischemic stroke | 3,971 | 8.5 | 8.1 | 0.01 |  | 8.3 | 8.2 | <0.01 |
| Chronic kidney disease | 3,971 | 21.9 | 28.7 | -0.15 |  | 25.8 | 28.1 | -0.05 |
| ESRD with dialysis | 3,971 | 2.9 | 5.6 | -0.12 |  | 5.7 | 5.2 | 0.02 |
| Laboratory data |  |  |  |  |  |  |  |  |
| NT-Pro BNP, pg/mL | 1,756 | 6327  [2771, 11500] | 5385  [2630, 10800] | -0.05 |  | 4883  [2148, 10070] | 5770  [2695, 10875] | -0.10 |
| ≥Median |  | 53.3 | 49.4 |  |  | 45.2 | 51.4 |  |
| <Median |  | 46.7 | 50.6 |  |  | 54.8 | 48.6 |  |
| Missing | 2,215 |  |  |  |  |  |  |  |
| Uric acid, mg/dL | 1,291 | 7.7 ± 4.5 | 7.5 ± 2.9 | 0.01 |  | 7.3 ± 3.1 | 7.5 ± 3.5 | -0.12 |
| Hyperuricemia (≥7) |  | 59.2 | 53.0 |  |  | 53.4 | 51.6 |  |
| Normal (<7) |  | 40.8 | 47.0 |  |  | 46.6 | 48.4 |  |
| Missing | 2,680 |  |  |  |  |  |  |  |
| Potassium (K), mEq/L | 3,103 | 4.1 ± 1.2 | 4.2 ± 0.7 | -0.05 |  | 4.2 ± 0.9 | 4.2 ± 0.8 | 0.03 |
| Hyperkalemia (≥5) |  | 7.3 | 7.5 |  |  | 7.6 | 7.6 |  |
| Normal (3.5-5) |  | 78.0 | 81.2 |  |  | 83.2 | 80.8 |  |
| Hypokalemia (≤3.5) |  | 14.7 | 11.3 |  |  | 9.3 | 11.6 |  |
| Missing | 868 |  |  |  |  |  |  |  |
| eGFR, mL/min/1.73m^2^ | 3,336 | 70.3 ± 67.4 | 64.7 ± 40.9 | 0.14 |  | 67.0 ± 50.6 | 65.0 ± 44.8 | 0.05 |
| <30 |  | 12.3 | 18.5 |  |  | 14.6 | 18.6 |  |
| 30-60 |  | 26.3 | 26.8 |  |  | 26.7 | 25.9 |  |
| ≥60 |  | 61.4 | 54.8 |  |  | 58.7 | 55.5 |  |
| Missing | 635 |  |  |  |  |  |  |  |
| Medication |  |  |  |  |  |  |  |  |
| MRA | 3,971 | - | - | - |  | 28.5 | 20.9 | 0.18 |
| RASI/ARNI | 3,971 | 45.0 | 35.7 | 0.19 |  | - | - | - |
| Beta-blocker | 3,971 | 35.6 | 28.2 | 0.16 |  | 36.5 | 27.4 | 0.20 |
| SGLT2i | 3,971 | 1.8 | 1.0 | 0.06 |  | 1.9 | 0.9 | 0.08 |
| Ivabradine | 3,971 | 1.3 | 0.6 | 0.07 |  | 1.2 | 1.1 | 0.01 |
| Loop diuretics | 3,971 | 45.6 | 37.2 | 0.17 |  | 47.3 | 37.4 | 0.21 |
| Thiazide | 3,971 | 8.1 | 5.8 | 0.09 |  | 8.3 | 5.4 | 0.12 |
| Antiplatelet | 3,971 | 15.6 | 12.0 | 0.16 |  | 13.2 | 12.4 | 0.20 |
| CCB | 3,971 | 20.1 | 19.9 | 0.01 |  | 23.8 | 18.3 | 0.14 |
| OHA* | 3,971 | 17.5 | 16.0 | 0.05 |  | 19.2 | 14.7 | 0.14 |
| Insulin | 3,971 | 15.6 | 14.0 | 0.04 |  | 12.9 | 14.5 | -0.04 |
| GLP1-RA | 3,971 | 0.17 | 0.15 | <0.01 |  | 0.2 | 0.3 | -0.03 |
| DOAC | 3,971 | 3.5 | 3.1 | 0.03 |  | 3.9 | 2.9 | 0.06 |
| Follow up duration, year | 3,971 | 3.6 ± 6.7 | 3.6 ± 4.0 | 0.01 |  | 3.7 ± 5.2 | 3.5 ± 4.2 | -0.03 |

Abbreviation: ARNI, angiotensin receptor neprilysin inhibitor; HFrEF, heart failure with reduced ejection fraction; GBM, generalized boosted modeling; IPTW, inverse probability treatment weighting; MRA, mineralocorticoid receptor antagonists; RASi, renin-angiotensin system inhibitor; STD, standardized difference; LVEF, left ventricular ejection fraction; COPD, chronic obstructive pulmonary disease; ESRD, end stage renal disease; NT-Pro BNP, N-terminal pro-brain natriuretic peptide; eGFR, estimated glomerular filtration rate; SGLT2i, sodium-glucose cotransporter 2 inhibitors; CCB, calcium channel blockers; OHA, oral hypoglycemic agent; GLP1-RA, glucagon-like peptide-1 receptor agonist; DOAC, direct oral anticoagulants;

* Not including SGLT2i;

Data are presented as frequency (percentage), mean ± standard deviation or median [25^th^, 75^th^ percentiles].

**Supplemental Table 6**. Follow up outcome of patients with HFrEF according to use of mineralocorticoid receptor antagonists or renin-angiotensin system inhibitor

|  | User | | Non-user | | HR/SHR | *P* |
| --- | --- | --- | --- | --- | --- | --- |
| Exposure / Outcome | Event rate (%) | Incidence (95% CI)# | Event rate (%) | Incidence (95% CI)# | (95% CI) | value |
| MRA |  |  |  |  |  |  |
| Efficacy outcome |  |  |  |  |  |  |
| Primary outcome* | 77.1 | 43.3 (41.6–45.0) | 72.1 | 46.1 (44.4–47.8) | 0.94 (0.89–0.99) | 0.014 |
| Cardiovascular death | 24.4 | 6.9 (6.4–7.4) | 24.2 | 6.7 (6.3–7.2) | 1.01 (0.92–1.11) | 0.77 |
| HHF | 68.1 | 38.2 (36.6–39.8) | 62.4 | 39.9 (38.3–41.5) | 1.03 (0.98–1.09) | 0.24 |
| Falsification endpoint |  |  |  |  |  |  |
| Psoriasis | 0.41 | 0.12 (0.05–0.18) | 0.34 | 0.10 (0.05–0.15) | 1.20 (0.57–2.55) | 0.63 |
| Influenza | 1.1 | 0.32 (0.21–0.42) | 1.0 | 0.28 (0.19–0.36) | 1.13 (0.72–1.77) | 0.61 |
| RASI/ARNI |  |  |  |  |  |  |
| Efficacy outcome |  |  |  |  |  |  |
| Primary outcome* | 72.5 | 40.8 (39.1–42.5) | 73.3 | 49.3 (47.5–51.1) | 0.86 (0.81–0.91) | <0.001 |
| Cardiovascular death | 22.9 | 6.2 (5.8–6.7) | 25.2 | 7.2 (6.7–7.6) | 0.88 (0.80–0.97) | 0.010 |
| HHF | 64.2 | 36.1 (34.5–37.7) | 63.3 | 42.6 (40.9–44.3) | 0.939 (0.887-0.995) | 0.035 |
| Falsification endpoint |  |  |  |  |  |  |
| Psoriasis | 0.45 | 0.12 (0.06–0.19) | 0.50 | 0.14 (0.08–0.21) | 0.91 (0.45–1.83) | 0.79 |
| Influenza | 0.73 | 0.20 (0.12–0.28) | 0.88 | 0.25 (0.17–0.34) | 0.83 (0.49–1.42) | 0.49 |

Abbreviation: ARNI, angiotensin receptor neprilysin inhibitor; IPTW, inverse probability treatment weighting; HFrEF, heart failure with reduced ejection fraction; CI, confidence interval; HR, hazard ratio; SHR, subdistribution hazard ratio; MRA, mineralocorticoid receptor antagonists; HHF, hospitalization for heart failure; CV, cardiovascular; RASi, renin-angiotensin system inhibitor;

* Composite of HHF and cardiovascular death;

# Number of events per 100 person-years.
