# Supplementary figures and images for: Comparative analysis of mineralocorticoid receptor antagonists and renin-angiotensin system inhibitors/angiotensin receptor neprilysin inhibitor in heart failure with mildly reduced ejection fraction
Source: Front Pharmacol. 2024 Dec 13;15:1507326. doi: 10.3389/fphar.2024.1507326 (PMC11671259; doi:10.3389/fphar.2024.1507326)

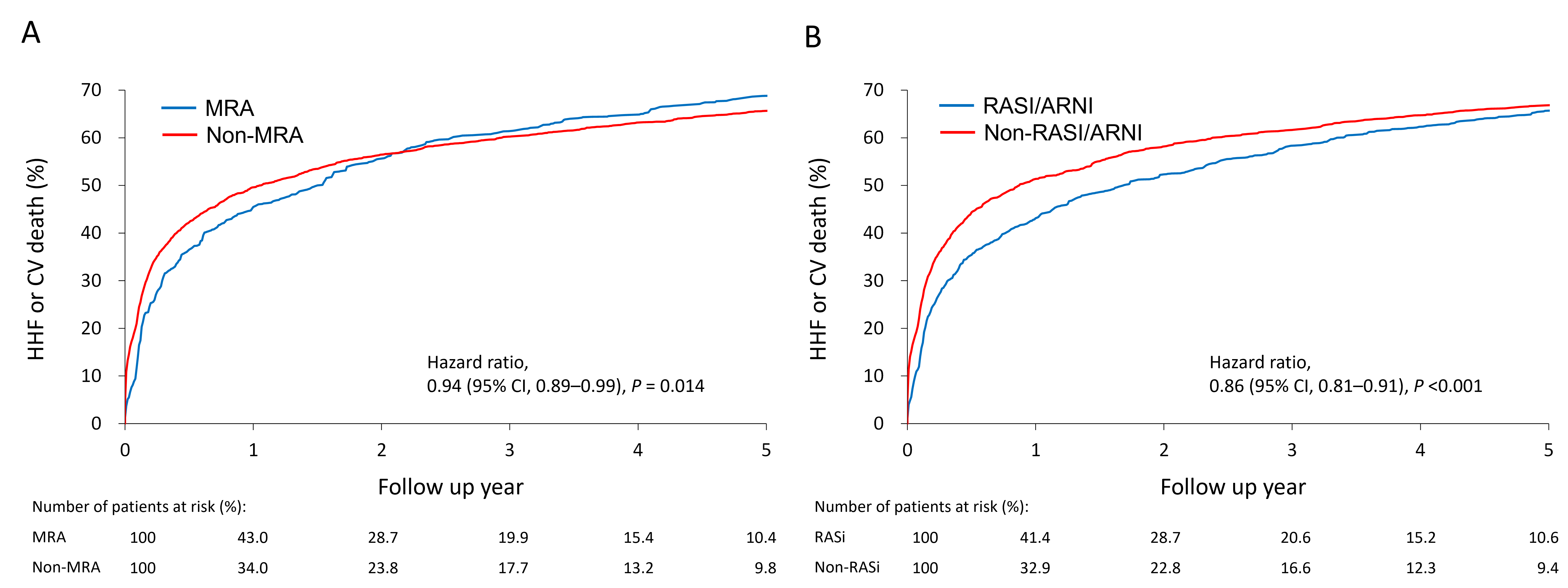

Supplement: Supplementary file 2 [file Image2.TIF]

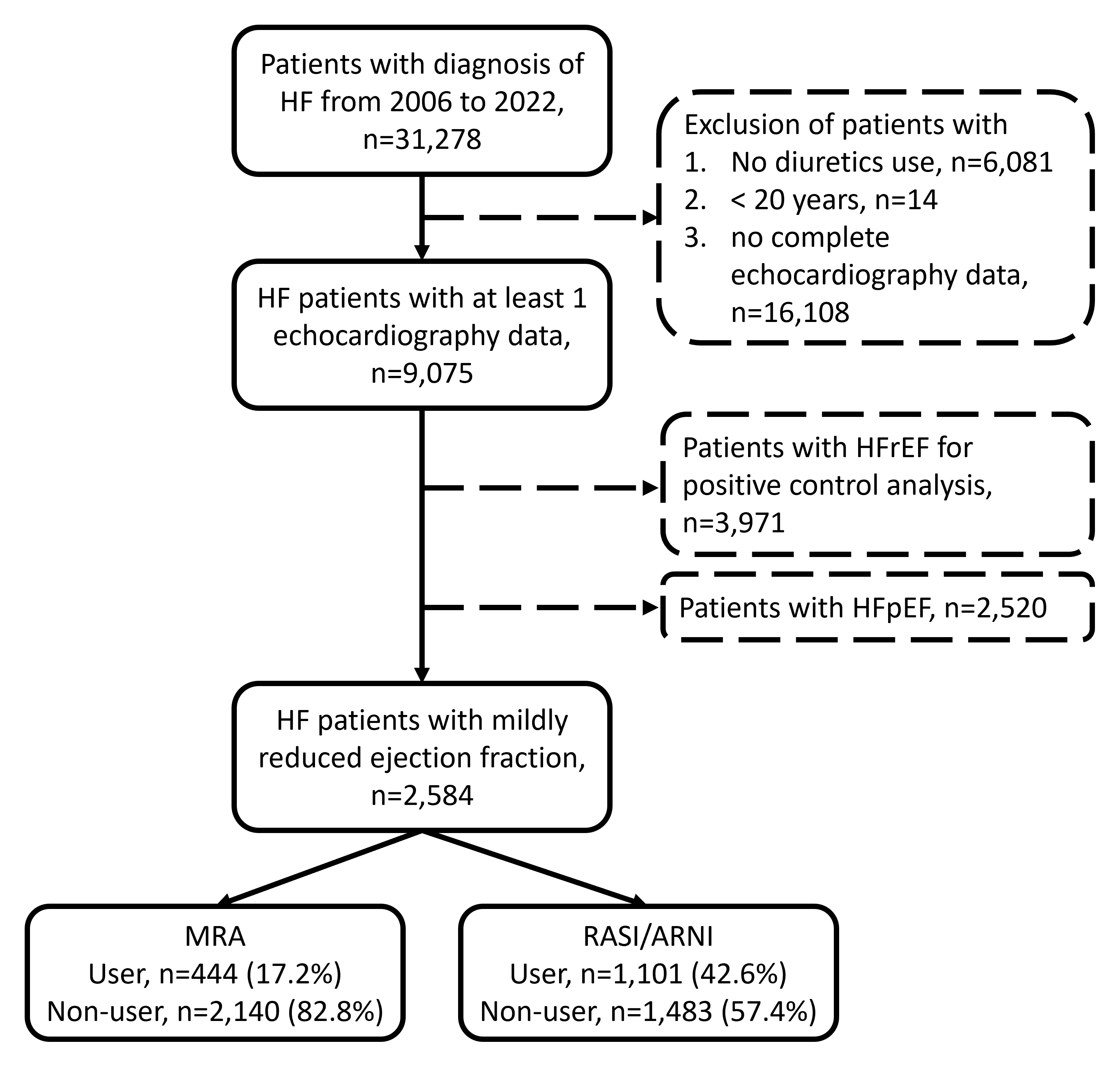

Supplement: Supplementary file 3 [file Image1.TIF]
